# Ribosomal Proteins RPS11 and RPS20, Two Stress-Response Markers of Glioblastoma Stem Cells, Are Novel Predictors of Poor Prognosis in Glioblastoma Patients

**DOI:** 10.1371/journal.pone.0141334

**Published:** 2015-10-27

**Authors:** William H. Yong, Maryam Shabihkhani, Donatello Telesca, Shuai Yang, Jonathan L. Tso, Jimmy C. Menjivar, Bowen Wei, Gregory M. Lucey, Sergey Mareninov, Zugen Chen, Linda M. Liau, Albert Lai, Stanley F. Nelson, Timothy F. Cloughesy, Cho-Lea Tso

**Affiliations:** 1 Department of Pathology and Laboratory Medicine, University of California Los Angeles, Los Angeles, California, United States of America; 2 Department of Biostatistics, University of California Los Angeles, Los Angeles, California, United States of America; 3 Department of Neurosurgery, General Hospital of Guangzhou Military Command, Guangzhou, China; 4 Department of Surgery/Surgical-Oncology, University of California Los Angeles, Los Angeles, California, United States of America; 5 Department of Human Genetics, University of California Los Angeles, Los Angeles, California, United States of America; 6 Department of Neurosurgery, University of California Los Angeles, Los Angeles, California, United States of America; 7 Department of Neurology/Neuro-Oncology, University of California Los Angeles, Los Angeles, California, United States of America; 8 Jonsson Comprehensive Cancer Center, David Geffen School of Medicine at University of California Los Angeles, Los Angeles, California, United States of America; Lawrence Berkeley National Laboratory, University of California, Berkeley, UNITED STATES

## Abstract

Glioblastoma stem cells (GSC) co-exhibiting a tumor-initiating capacity and a radio-chemoresistant phenotype, are a compelling cell model for explaining tumor recurrence. We have previously characterized patient-derived, treatment-resistant GSC clones (TRGC) that survived radiochemotherapy. Compared to glucose-dependent, treatment-sensitive GSC clones (TSGC), TRGC exhibited reduced glucose dependence that favor the fatty acid oxidation pathway as their energy source. Using comparative genome-wide transcriptome analysis, a series of defense signatures associated with TRGC survival were identified and verified by siRNA-based gene knockdown experiments that led to loss of cell integrity. In this study, we investigate the prognostic value of defense signatures in glioblastoma (GBM) patients using gene expression analysis with Probeset Analyzer (131 GBM) and The Cancer Genome Atlas (TCGA) data, and protein expression with a tissue microarray (50 GBM), yielding the first TRGC-derived prognostic biomarkers for GBM patients. Ribosomal protein S11 (RPS11), RPS20, individually and together, consistently predicted poor survival of newly diagnosed primary GBM tumors when overexpressed at the RNA or protein level [RPS11: Hazard Ratio (HR) = 11.5, p<0.001; RPS20: HR = 4.5, p = 0.03; RPS11+RPS20: HR = 17.99, p = 0.001]. The prognostic significance of RPS11 and RPS20 was further supported by whole tissue section RPS11 immunostaining (27 GBM; HR = 4.05, p = 0.01) and TCGA gene expression data (578 primary GBM; RPS11: HR = 1.19, p = 0.06; RPS20: HR = 1.25, p = 0.02; RPS11+RPS20: HR = 1.43, p = 0.01). Moreover, tumors that exhibited unmethylated O-6-methylguanine-DNA methyltransferase (MGMT) or wild-type isocitrate dehydrogenase 1 (IDH1) were associated with higher RPS11 expression levels [corr (IDH1, RPS11) = 0.64, p = 0.03); [corr (MGMT, RPS11) = 0.52, p = 0.04]. These data indicate that increased expression of RPS11 and RPS20 predicts shorter patient survival. The study also suggests that TRGC are clinically relevant cells that represent resistant tumorigenic clones from patient tumors and that their properties, at least in part, are reflected in poor-prognosis GBM. The screening of TRGC signatures may represent a novel alternative strategy for identifying new prognostic biomarkers.

## Introduction

Glioblastoma (GBM) WHO grade IV is the most common and aggressive brain tumor in adults, and currently has no cure. Temozolomide (TMZ), administered concurrently with and without radiation therapy (RT), is the standard first-line treatment in GBM [1–4]. Methylation of the O-6-methylguanine-DNA methyltransferase (MGMT) promoter has emerged as an important prognostic and predictive factor for TMZ treatment of newly diagnosed GBM [5]. Likewise, mutations in isocitrate dehydrogenase (IDH) predict a good prognosis, and are more frequently seen in secondary GBM [6]. However, despite the best efforts of gross total resection and post-operative radiochemotherapy, most patients still develop tumor recurrence. The overall 5-year survival rate is lower than 10%, and the 10-year mortality rate is nearly 100% [3]. Bevacizumab, an anti-angiogenic drug, was designed to block vascular endothelial growth factor (VEGFA), and is FDA-approved as the second-line therapy for treating recurrent GBM. However, while improving progression-free survival, the addition of bevacizumab to RT-TMZ did not improve survival in patients with glioblastoma [7, 8, 9]. Moreover, the rate of adverse events was actually higher with bevacizumab than with placebo [8, 9]. Thus, identification of novel treatment targets associated with patient prognosis for GBM remains a highly important goal.

Glioblastoma stem cells (GSC) or stem-like glioblastoma-initiating cells (GIC) are a significant preclinical model for explaining and testing the mechanisms underlying post-treatment tumor recurrence, because these patient tumor-derived cells exhibit a tumorigenic capacity [10–15], a highly migratory nature [15–16], and a radio-chemoresistant phenotype [17–19]. We previously reported that neurosphere formation is an independent predictor of glioma tumor progression independent of Ki67 proliferation index and suggested that the ability to propagate GSC in vitro is associated with clinical outcome [20]. These findings lend support to the view that upregulation of GSC-associated properties and activity in tumors may be associated with poor prognosis in GBM patients. Although CD133 is not a defining marker of GSC, the CD133 antigen has been identified as a putative stem cell marker in normal and malignant brain tissues and was the first surface marker utilized for the enrichment of GSC [10, 11, 13, 15]. The prognostic value of CD133 in GBM has been evaluated by several independent groups [21–23]. The proportion of CD133^+^ cells in tumor tissues is an independent risk factor for tumor regrowth and for time to malignant progression [21]. Moreover, high expression levels of CD133 is associated with a shorter time to recurrence at locations distant from the original site [22] and with higher grades of glioma as well as a worse prognosis [23]. More recently, LGR5 (leucine-rich repeat-containing G protein-coupled receptor 5), a novel stem cell marker of the intestinal epithelium and the hair follicle, was reported to be a poor prognostic factor in GBM and to be required for survival of glioblastoma stem-like cells [24]. Interestingly, we previously found that upregulated LGR5 is a shared marker of CD133^+^ GSC when compared to CD133^-^GSC [15] and upregulation of LGR5 was also detected in GSC that survived high-dose TMZ treatment when compared to untreated parental GSC (unpublished data). These findings suggested that upregulated genes in GSC with a stress-resistant phenotype might be a good resource for identifying novel biomarkers that predict the outcome of GBM patients.

Previously, to explore the potential mechanisms underlying treatment resistance of GBM, we isolated and characterized tumorigenic GSC clones that survived radiochemotherapy [25]. We found that, even under glucose-containing culture condition supplemented with insulin, these quiescent treatment-resistant GSC clones (TRGC) expressed a glucose restriction (GR)-like phenotype, and used lipid catabolism pathways in mitochondria as their primary energy source, whereas their autologous treatment–sensitive GSC clones (TSGC) were highly dependent on glycolysis for ATP production [25]. Functionally, TRGC upregulate CD133/SOX2, and exhibit increased autophagic activity and AMP-activated protein kinase (AMPK)-SIRT1 signaling. Additionally, upregulated genes are associated with global DNA repair activity, pointing to a metabolic stress-induced protective response for stabilizing cellular, molecular and genomic integrity [26–33]. Concurrently, the molecular defense signatures of TRGC identified via comparative expression microarray analysis resemble anti-aging and anti-stress effects of a GR-like phenotype [34, 35]. The siRNA-based on-target gene knockdown experiments further confirmed the role of selected defense signatures in stress-resistance of TRGC [25] and suggested that the molecular response programs essential for self-defense in TRGC are likely to at least partially contribute to the development of treatment resistance and tumor recurrence.

In this study, we extend our prior work and test the hypothesis that defense signatures upregulated in TRGC are able to serve as prognostic factors for GBM patients. We screened a subset of TRGC signatures in GBM surgical samples and The Cancer Genome Atlas (TCGA) dataset, and have identified novel TRGC-derived biomarkers predictive of poor prognosis in patients with newly diagnosed GBM.

## Materials and Methods

### Probe Set Analyzer correlation of gene expression with GBM patient survival curves

To assess the correlation of transcriptional levels of defense signatures in patient tumors with patient survival, Probe Set Analyzer, a free publicly available, web-based interactive tool for real time correlation of gene expression values and patient survival was employed. The tool was created and developed through a collaboration between UCLA Neuro-Oncology, UCLA Human Genetics and SiliconMED (http://probesetanalyzer.com). The arrays and survival data were derived from consented patients treated by the UCLA Neuro-Oncology program. The expression microarray data consists of 22,000 probe sets analyzed on 131 GBM, including 67 newly diagnosed and 64 recurrent GBM. Recurrent GBM are cases where tumors were previously diagnosed as GBM, whether primary or secondary, and recurred. In neurooncology terminology, primary GBM are defined as tumors that present at first diagnosis as GBM, without apparent evolution from a low grade glioma [36]. Secondary GBM, in contrast, are designated as such if the patient first presented with a lower grade glioma (Grade II or Grade III) and subsequently transformed into a GBM WHO Grade IV. The Probe Set Analyzer dataset does not distinguish primary from secondary GBM. The Probe Set Analyzer generated Kaplan-Meier curves for different gene expression levels (Low, Mid, High) and also determined whether the curves were statistically significant providing a Log-rank test p-value.

### Immunohistochemical analysis of a glioblastoma tissue microarray (TMA)

A GBM tissue microarray was constructed consisting of three representative 1-mm cores from formalin-fixed, paraffin-embedded (FFPE) tissue blocks from each one of 50 GBM patients. All GBM tumor specimens were obtained from patients who underwent surgery at Ronald Reagan UCLA Medical Center. All samples collected were under patients’ written consent, and were approved by the UCLA Institutional Review Board. Twenty-eight were newly diagnosed GBM and 22 were recurrent GBM. 21 of the newly diagnosed GBM were primary GBM and 7 were secondary GBM. Eighteen recurrent GBM were primary GBM and 4 were secondary GBM. Immunohistochemical analysis was performed on 5-μm FFPE sections of TMAs. Antigen retrieval was performed by immersing TMA slides into a 1X Dako Target Retrieval buffer for 40 min in a pressure cooker. Endogenous peroxidase activity was quenched with 3% hydrogen peroxide in PBS. TMA sections were incubated with diluted primary antibodies ribosomal protein S11 (RPS11) at 1:25, ribosomal protein S20 (RPS20) at 1:100, Vascular endothelial growth factor A (VEGFA) at 1:50 (Prestige Antibodies®, Sigma, St. Louis, MO) for 16 h at 4°C, followed by incubation with MACH-3 (Rabbit) or MACH-4 (Mouse) secondary antibodies (Biocare Medical, Concord, CA) for 30 minutes at room temperature. Subsequent immunodetection was completed using Vector NovaRed (Vector Laboratories, Burlingame, CA).

### Immunohistochemical staining of whole tissue sections of newly diagnosed primary glioblastoma

Five micrometer thick-whole tissue sections were cut from paraffin blocks of 27 newly diagnosed GBM. These cases had available MGMT methylation-specific polymerase chain reaction (PCR) and IDH1 immunohistochemistry or PCR results. Thirteen cases of these whole tissue sections were of tumors previously represented on the TMA and had adequate tissue for further study. Many cases had limited tissue after three cores were obtained for the TMA and were not suitable for whole tissue section immunostaining. Another 14 cases were additional newly diagnosed GBM not on the TMA. Immunostaining was performed as described above**.**

### Scoring and interpretation of immunohistochemistry

Tissue microarray sections were separately stained with each selected antibody. Individual antibody stains were screened by a pathologist (W.Y.) for variability of staining intensity between cases on the premise that a high degree of variability between GBM cases would permit more robust correlation with survival than stains that showed scant differences between cases. Immunohistochemistry for these immunostains were scored semi-quantitatively. Intensity of staining was scored on a scale of 0–3 (0 = no staining; 1 = weak; 2 = moderate; 3 = strong). The percentage of tumor cells expressing each level of intensity was also scored. An H score (range = 0–300) for each stained tumor was generated by multiplying the percentages by their intensity score and adding up the products of multiplication [37, 38].

### Statistical analysis for immunohistochemistry using Cox Proportional Hazard Analysis (Pairwise analyses- Optimal binning)

**Fig 1:**
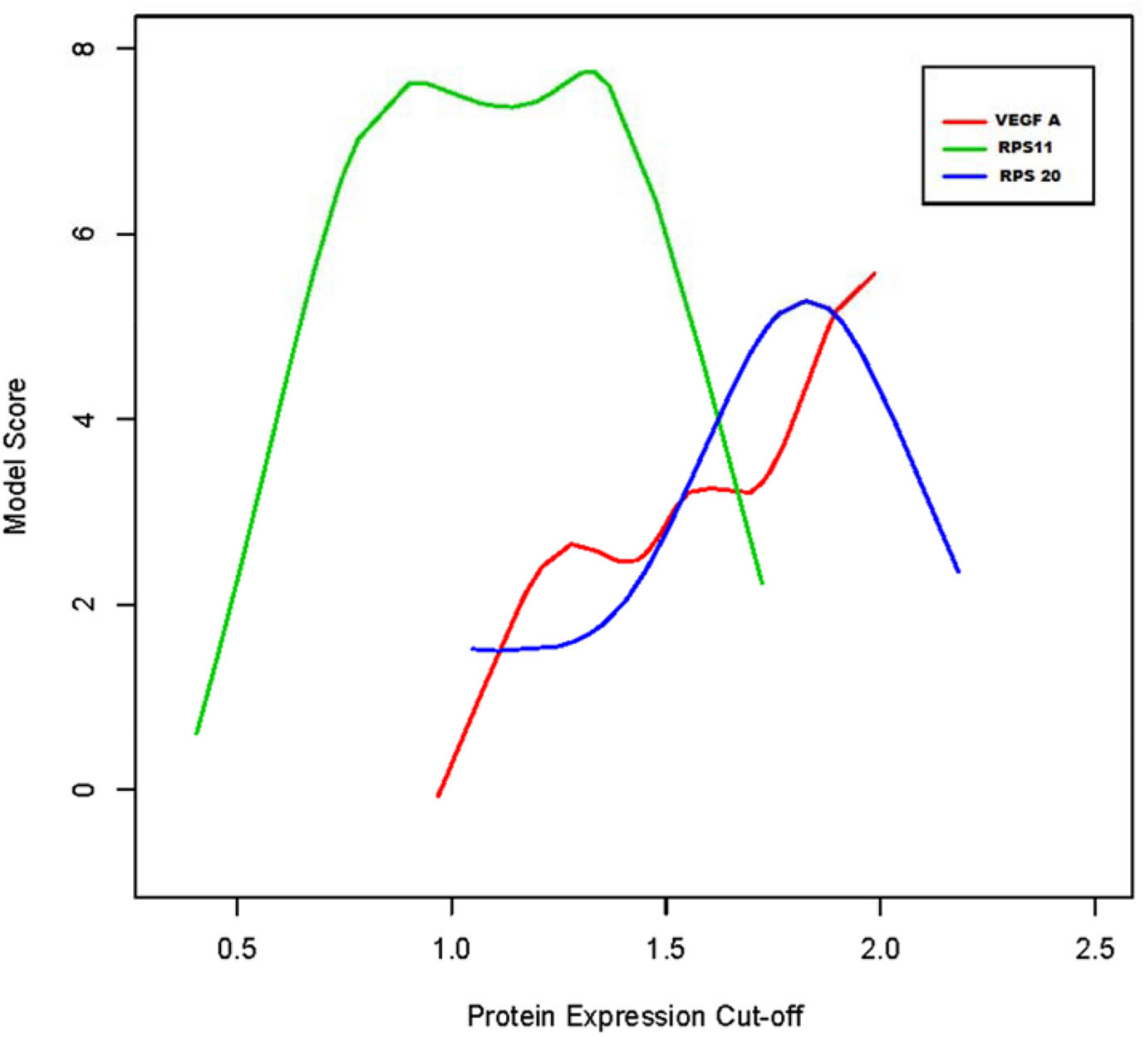
Modeling of protein expression (H-score) cutoffs for low vs. high protein expression. Each curve reports the score of a Cox Proportional Hazards model associated with different cut-off values of the related protein expression to bin scores into Low versus High expression. Protein expression is measured as the H score rescaled to (0–3). Optimal cut-offs are identified as the mode in the cut-off vs. score curve. For VEGFA, this was not identifiable and an 80^th^ percentile H-score was used as a cut-off.

The H-scores for RPS11, RPS20, and VEGFA were assessed for association with survival. Survival was defined from the first day of surgery to the last day of follow up or death. We investigated if an H-score specific for each of the 3 proteins could be found to best discriminate between patients’ prognostic outcomes. For each protein, we considered several possible average expression values to define high versus low expression groups. For each grouping scenario, we fitted a Cox Proportional Hazards model and computed the model score (Fig 1). The model with the highest score should indicate the protein-specific expression cut-off that best discriminates between patients with poor and best survival. For VEGFA, the 80^th^ percentile was used as modeling did not identify optimal binning. The determined optimal cutoffs were 1.80 (VEGFA), 1.33 (RPS11), and 1.83 (RPS20) respectively. Association of protein expression with all GBM, primary GBM, secondary GBM, newly diagnosed primary GBM and recurrent GBM with survival were then analyzed with Cox Proportional Hazards (CPH) Analysis. In our CPH analysis we consider both binned (high vs. low) protein expression and continuous expression scores as potential predictors of survival. Differences in the death hazard were estimated as HR in high vs. low expression groups. Our conclusions did not change, qualitatively, when we considered continuous expression scores. The proportional hazard assumption was evaluated inspecting Kaplan Meyer estimates of survival functions in the high vs. low expression groups. The CPH model was found adequate to describe differential survival. Mean protein expression of RPS11 (whole tissue sections of newly diagnosed GBM, n = 27) in MGMT methylated GBM patients was compared against MGMT unmethylated patients using a simple T-test. Similarly, mean expression of RPS11 in IDH1 mutant GBM patients was compared against IDH1 wild type patients using a simple T-test.

### Gene expression levels correlated with survival from The Cancer Genome Atlas data

The TCGA (https://tcga-data.nci.nih.gov/tcga/) data repository was used to further validate our findings in 578 newly diagnosed primary GBM patients. In the TCGA database, protein expression in GBM was assessed with a reverse phase protein array (RPPA) platform and mRNA gene expression measurements were obtained using Agilent Expression 244K microarrays. The GBM proteome data in the TCGA database was limited to 171 proteins and none of the proteins of interest (RPS11, RPS20, and VEGFA) was included among them. We therefore focused on analysis of TCGA gene expression data. The response of interest was survival after diagnosis. We considered average normalized gene expression as a continuous predictor of survival, where the baseline hazard refers to subjects with average expression. The HR is to be interpreted as the expected change in the hazard of death as expression increases by 1 standard deviation. A binned analysis was also performed, where we compared subjects with gene expression above average vs. subjects with gene expression below average.

## Results

### Probe Set Analyzer analysis shows that elevated transcription levels of TRGC-associated genes in GBM tissues have a negative correlation with patient survival

**Fig 2:**
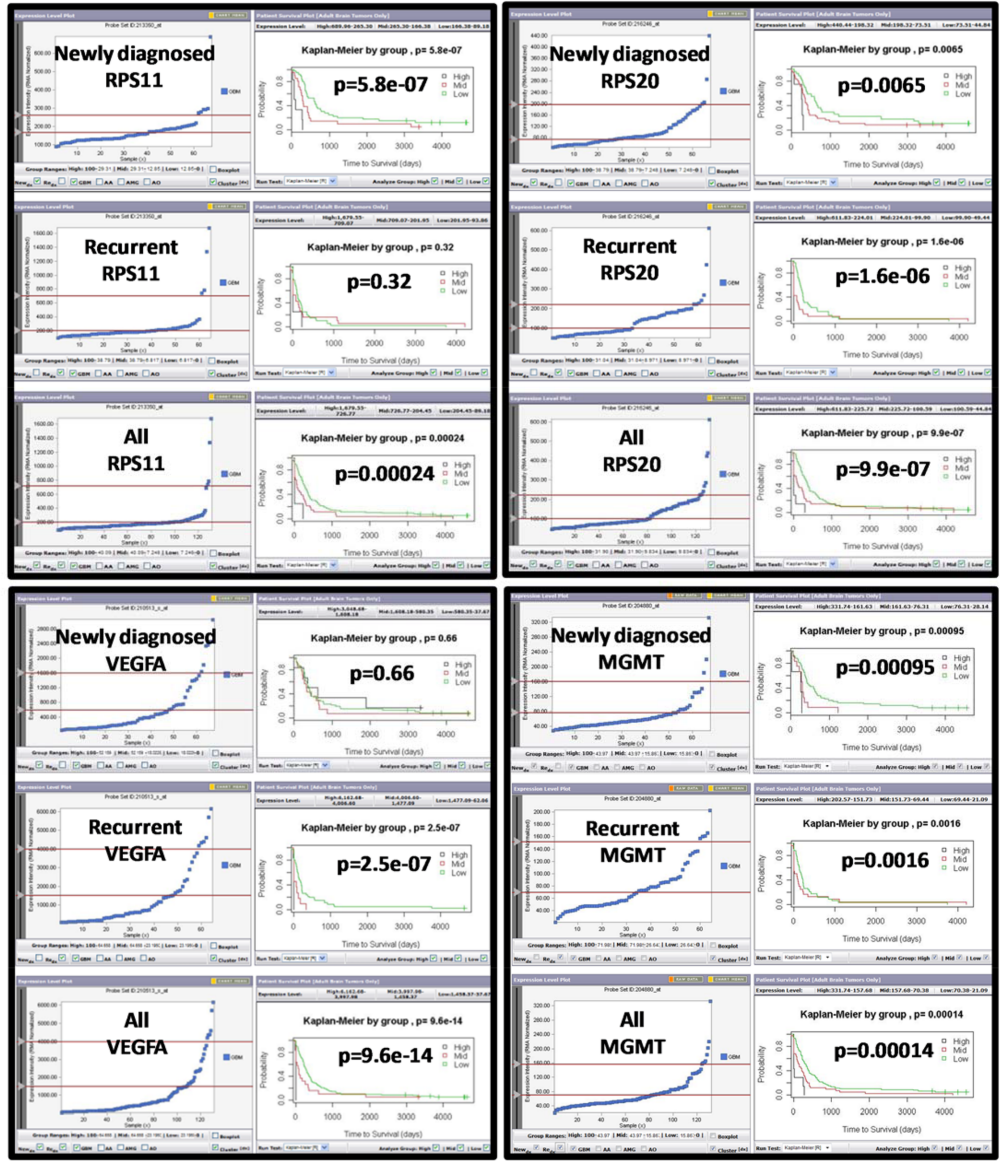
Representative outputs of Probe Set Analyzer for selected molecular signatures of TRGC. A real-time correlation of patient survival and gene expression levels of selected signatures of TRGC was performed using Probe Set Analyzer (http://probesetanalyzer.com/index.aspx). “Newly Diagnosed", “Recurrent" or “combined all tumors” were analyzed. The y-axis represents normalized gene expression intensity, and the x-axis represents patient groups. The sliding bars position patients into high, mid and low gene expression level groups. A Kaplan-Meier curve with corresponding p-values is generated based on each change to these patient groups. An integrated statistical engine calculated p-values based on Kaplan-Meier curves.

We have identified defense signatures of TRGC via comparative analysis of expression profiles of TSGC and TRGC [25]. Since both populations are stem-like, tumor-initiating cells and may only differ in their degree of differentiation, we anticipate that the defense signatures of TRGC will be mainly associated with stress-resistant phenotype. We therefore hypothesized that defense signatures of TRGC would be an excellent resource for identifying prognostic markers in GBM patients. To test this hypothesis, we firstly employed Probe Set Analyzer to screen 53 defense signatures of TRGC. We found all 53 TRGC signatures could be detected in patient tumors as indicated by Probe Set Analyzer. The association of transcriptional levels of TRGC signatures with patients’ survival was identified either in newly diagnosed tumors, recurrent tumors, or both (S1 Table). We found 9 markers to be significantly associated with poor prognosis when expression levels were upregulated only in newly diagnosed tumors, while 12 markers were associated with shorter survival when upregulated only in recurrent tumors. We also identified 7 markers that had prognostic value in both newly diagnosed and recurrent tumors. Even though the MGMT promoter status, rather than the MGMT expression, is important as a marker to predict therapy resistance, we found several TRGC markers to be better predictors of patient survival than MGMT gene expression as indicated by the Probe Set Analyzer gene expression data (Fig 2, S1 Fig). This data raises the possibility that subpopulations or populations of tumor cells in a GBM either convergently use or may have inherited stress resistance pathways from TRGC contributing to the development of treatment resistance.

### Elevated ribosomal protein levels in newly diagnosed GBM and primary GBM predict poor prognosis

**Fig 3:**
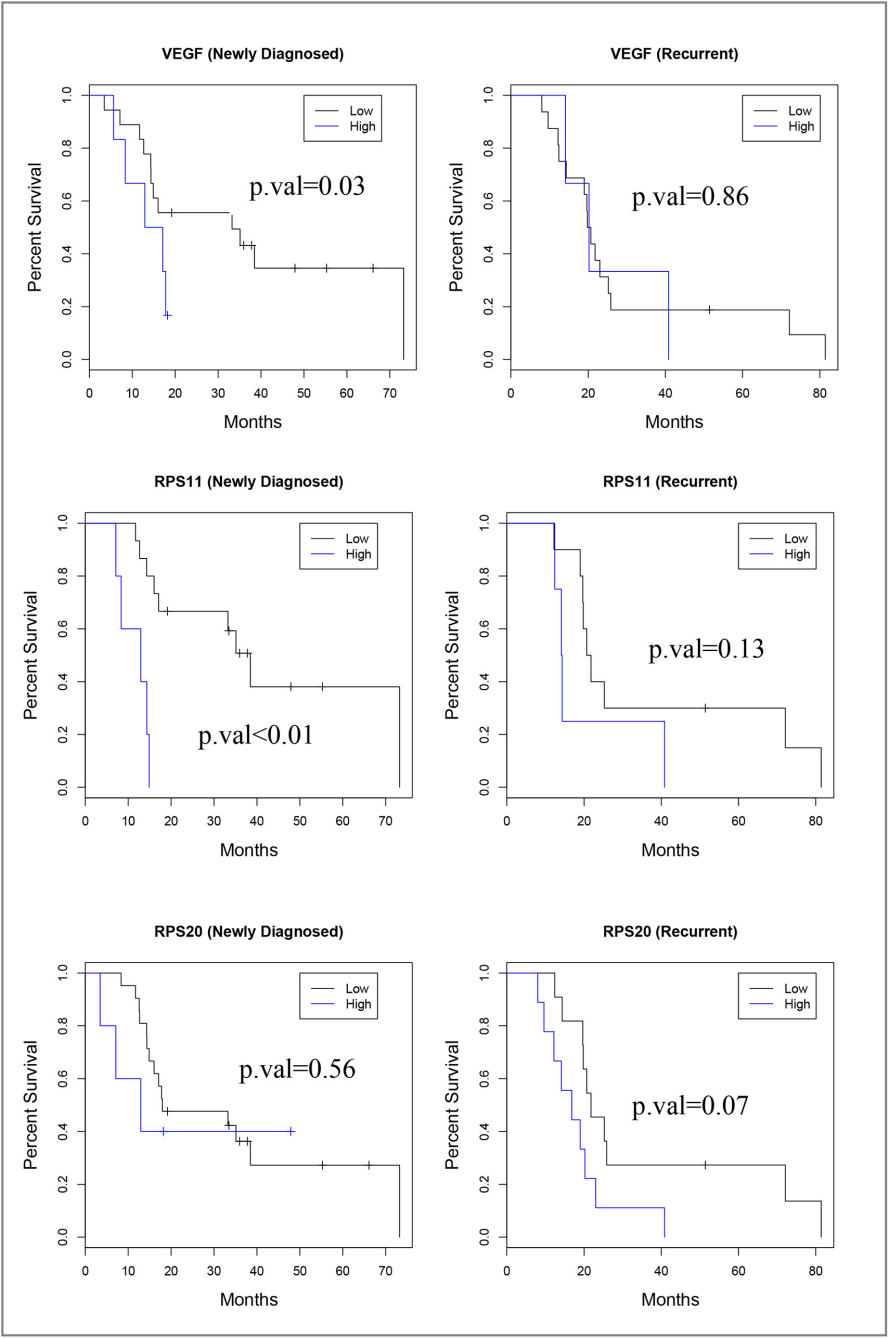
Kaplan-Meier survival curves for newly diagnosed and recurrent GBMs expressed differential levels of selected signatures of TRGC. These survival curves for RPS11, RPS20, and VEGFA correspond to the Cox Proportional Hazards data in Table 1. Both primary and secondary GBMs were included in the analyses.

**Fig 4:**
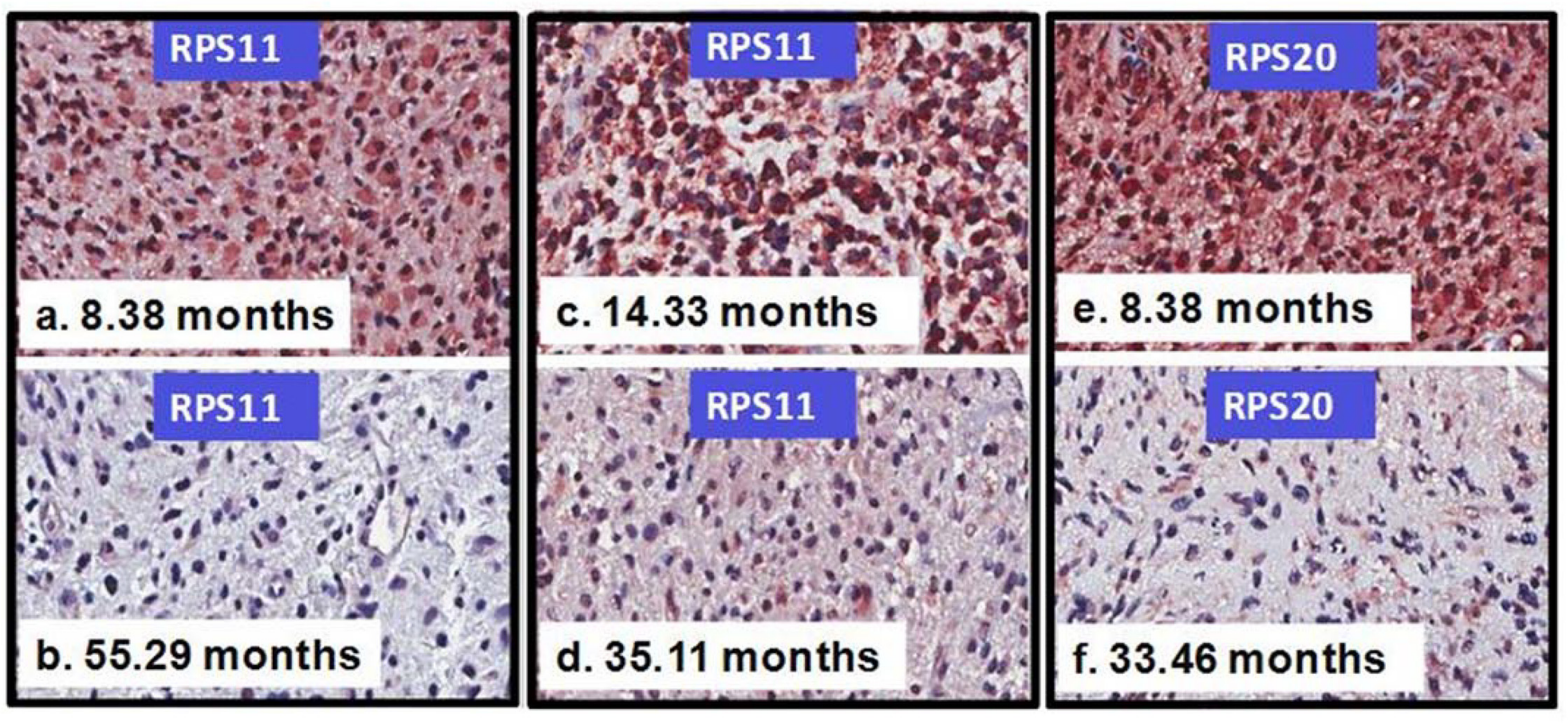
Upregulation of protein levels of TRGC signatures in GBM subgroups correlates with patients' poor prognosis. Representative immunohistochemical staining of RPS11 and RPS20 in clinical GBM subgroups. Upregulation of RPS11 was found to increase the hazard of death (HOD) 11-fold in newly diagnosed primary GBM (a, b) (Table 2) and 7-fold in a secondary GBM (c, d) (S2 Table). Upregulation of RPS20 was found to increase the HOD 5-fold in newly diagnosed primary GBM (e, f) (Table 2). The survival times of each representative patient are shown.

Based on gene function annotations, siRNA knockdown studies [25], and Probe Set Analyzer data (Fig 2, S1 Table, S1 Fig), immunostaining of 10 selected markers was performed on sections of the GBM tissue microarray. Six markers (FMNL2, PRKCI, FOLR1, OSBPL8, PPP1R3C, and MFAP4) (Full names of these genes are shown in S1 Table) showed relatively uniform and strong immunohistochemical staining across cases and were not further evaluated (data not shown). One marker (IL6ST) showed consistently weak staining across tumors and also was not further studied (data not shown). Three markers, RPS11, RPS20, and VEGFA, showed a range of staining among samples and were subjected to detailed scoring. H-scores were tabulated and subjected to statistical analysis. When all GBM were analyzed (Table 1), high RPS11 expression was associated with a 4.3-fold increased likelihood of death, whether a CPH binned (Table 1) or continuous score methodology (data not shown) was used. High RPS20 expression showed a 2-fold increased likelihood of death only when using the continuous score methodology (data not shown) and approached significance with a binned methodology. The whole GBM dataset was further subdivided into newly diagnosed or recurrent GBM (Table 1, Fig 3). Upregulation of VEGFA and RPS11 was found to increase the hazard of death 3.5-fold and 9-fold in newly diagnosed tumors only respectively. In age-adjusted analyses, results remain substantially unchanged (Table 1). In evaluating primary GBMs, upregulation of RPS11 was found to increase the hazard of death 4-fold and upregulation of RPS20 was found to increase the death hazard by 2.6-fold (Table 2). For primary GBM patients, upregulation of VEGFA did not change the hazard of death significantly (Table 2). Moreover, increased VEGFA and RPS11 expression, but not RPS20, in secondary GBM was associated with poor prognosis (S2 Table). The primary GBM dataset was subdivided into newly diagnosed and recurrent cohorts and analyzed with CPH analysis (Table 2). VEGFA expression was not associated with survival in either recurrent or newly diagnosed tumors. Upregulation of RPS11 and RPS20 was found to increase the hazard of death 11-fold and 4.5-fold, respectively, in newly diagnosed primary GBM. In newly diagnosed primary GBM, the HR for patients with overexpressed proteins RPS11 or RPS20 was 1.98 (95% C.I. 0.52–7.48, p = 0.32), when compared to patients with low RPS11 or RPS20. The HR for patients with simultaneous overexpression of both proteins RPS11 and RPS20 was 17.99 (95% C.I. 3.01–107.55, p = 0.001) when compared to patients with low RPS11 and RPS20 (Table 2). In age-adjusted analyses, results remain substantially unchanged (Table 2). Representative immunohistochemical staining of RPS11 and RPS20 in clinical GBM subgroups is presented in (Fig 4). When overexpressed, RPS11 and RPS20 were generally diffusely upregulated in the tumor cell population. However, we found that RPS11 was the only one of the 3 biomarkers where an upregulated gene expression profile (determined by Probe Set Analyzer) was completely concordant with increased protein expression in terms of increasing the hazard of death for newly diagnosed GBM (Table 3). Given the consistent protein and gene expression data in RPS11, we extended evaluation of RPS11 to whole tissue sections of newly diagnosed GBM. In whole tissue sections, we observed a 4-fold increase in the hazard of death, when comparing patients with high expression levels of RPS11 (expression > 1.33) to patients with low RPS11 expression levels (HR = 4.05, 95% C.I. = 1.39–11.78, p = 0.01). Similar results were obtained considering raw protein expression scores as predictors of survival.

**Table 1:** Tissue microarray protein expression- Cox proportional hazard analysis (primary and secondary GBMs included).

Biomarker	All GBMs	Newly Diagnosed	Recurrent HR (95%CI) [p value]
	HR (95%CI) [p value]	HR (95%CI) [p value]	
**RPS11** (Expression > 1.3)	4.34 (1.82–10.34) [<0.01]	9.29 (2.61–33.14) [<0.01]	2.61 (0.92–7.38) [0.13]
**RPS20** (Expression > 1.8)	1.98 (0.97–4.02) [0.06]	1.45 (0.42–5.06) [0.56]	2.36 (1.06–5.23) [0.07]
**VEGFA** (Expression > 1.8)	1.97 (0.98–3.96) [0.06]	3.46 (1.13–10.61) [0.03]	1.12 (0.39–3.21) [0.86]
**Age-adjusted estimates**			
**RPS11** (Expression > 1.3)	3.92 (1.88–8.17) [<0.01]	5.61 (1.92–16.41) [<0.01]	2.37 (0.82–6.82) [0.18]
**RPS20** (Expression > 1.8)	1.93 (1.06–3.53) [0.07]	2.73 (0.93–8.06) [0.13]	1.15 (0.47–2.83) [0.80]
**VEGFA** (Expression > 1.8)	1.30 (0.63–2.67) [0.55]	1.69 (0.63–4.49) [0.38]	0.78 (0.26–2.28) [0.70]

**Table 2:** Tissue microarray protein expression- Cox proportional hazard analysis (primary GBMs only).

Biomarker	All GBMs	Newly Diagnosed	Recurrent
	HR (95%CI) [p value]	HR (95%CI) [p value]	HR (95%CI) [p value]
**RPS11** (Expression > 1.3)	3.95(1.52–10.28) [0.005]	11.46 (2.71–48.33) [<0.01]	1.45 (0.37–5.65) [0.65]
**RPS20** (Expression > 1.8)	2.58 (1.20–5.58) [0.02]	4.51 (1.19–17.10) [0.03]	2.26 (0.94–5.46) [0.13]
**VEGFA** (Expression > 1.8)	1.47 (0.55–3.94) [0.44]	2.83 (0.72–11.15) [0.14]	0.76 (0.21–2.72) [0.73]
**RPS11 and RPS20**	4.09 (1.26–13.26) [0.02]	17.99 (3.01–107) [0.001]	NA
**Age-adjusted estimates**			
**RPS11** (Expression > 1.3)	3.85 (1.64, 9.01) [<0.01]	6.82 (2.05–22.66) [<0.01]	1.50 (0.37–6.14) [0.63]
**RPS20** (Expression > 1.8)	2.06 (0.94–4.56) [0.07]	3.94 (1.28–12.17) [0.049]	0.96 (0.34–2.75) [0.95]
**VEGFA** (Expression > 1.8)	0.98 (0.42–2.31) [0.97]	1.39 (0.43–4.46) [0.65]	0.46 (0.13–1.70) [0.33]
**RPS11 and RPS20**	5.84 (1.64–20.8) [<0.01]	19.9 (2.65–251) [0.001]	NA

**Table 3:** Correlation with poor survival in newly diagnosed and recurrent GBMs (primary and secondary GBMs included).

Biomarker(s)	Gene expression		Protein expression	
	Probe Set Analyzer		Tissue microarray IHC- GBM	
	Newly diagnosed	Recurrent	Newly diagnosed	Recurrent
	(p value)	(p value)	HR (P value)	HR (P value)
**RPS11**	<0.001	0.32	9.29 (<0.001)	2.61 (0.13)
**RPS20**	0.0065	<0.001	1.45 (0.56)	2.36 (0.07)
**VEGFA**	0.66	<0.001	3.46 (0.03)	1.12 (0.86)

### Elevated RPS11 protein expression is associated with unmethylated MGMT status or wild-type IDH1 status

**Fig 5:**
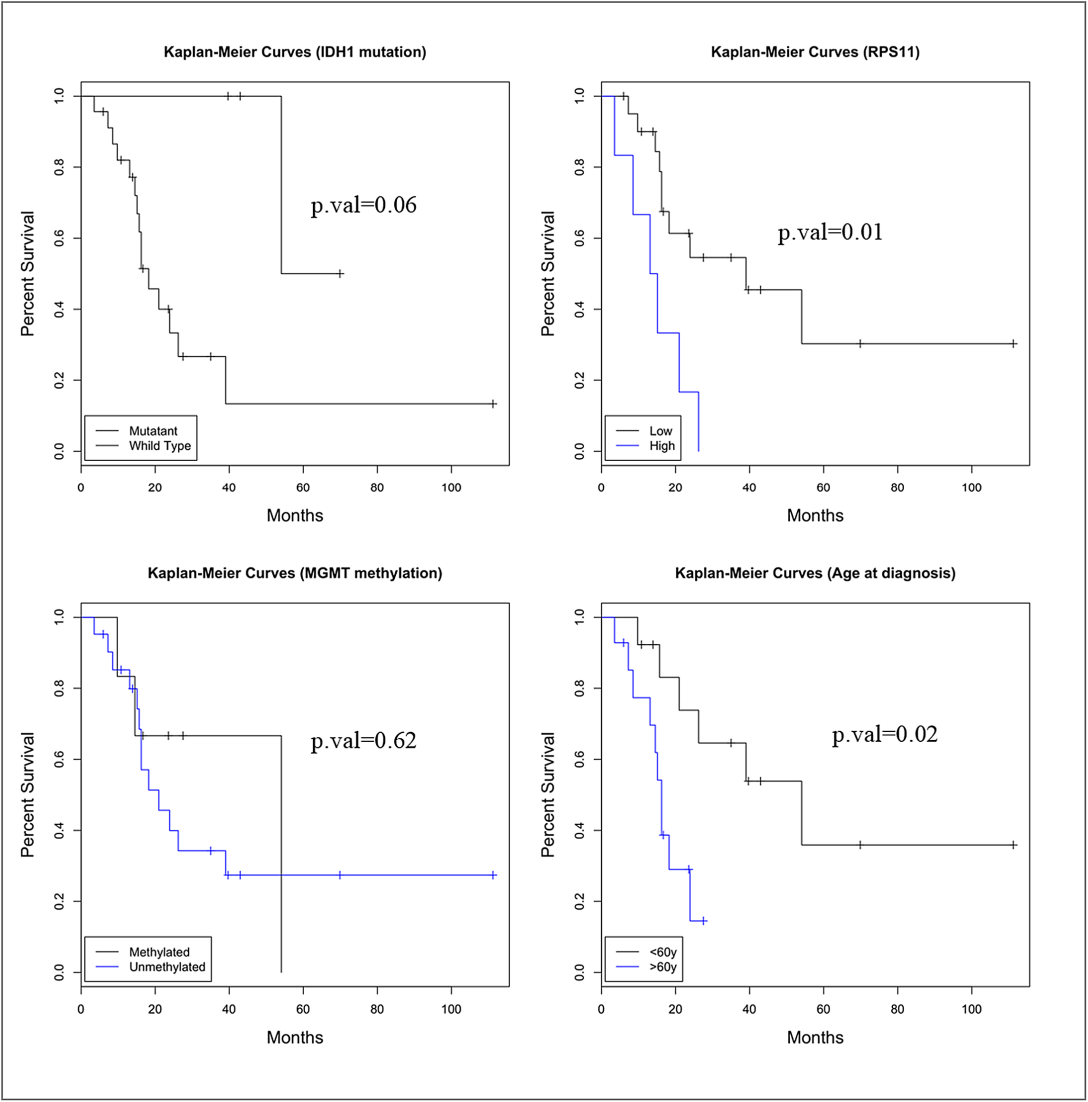
Correlations of the expression level of RPS11, the status of MGMT methylation, IDH mutation and age with patient survival. Each indicated factor was analyzed as an independent prognostic factor. Changes in relative risk were depicted with Kaplan-Meier curves and analyzed using log-rank tests.

MGMT unmethylated status and wild-type IDH1 status are individually associated with poor prognosis [1, 39], and the IDH1 mutation is positively associated with an MGMT methylated status [40]. Using the 27 cases with whole tissue sections, we also evaluated the association of RPS11 protein levels with MGMT and IDH1 status. MGMT was methylated in 22.2% and IDH1 was mutated in 14.8% of these patients. We estimated the difference in average RPS11 expression between patient subgroups. Patients with unmethylated MGMT exhibited higher RPS11 expression levels (mean difference = 0.52, 95% C.I. 0.05–0.99, p = 0.04), when compared to patients with methylated MGMT. Patients with wild-type IDH1 exhibited higher RPS11 expression levels (mean difference = 0.64, 95% C.I. 0.09–1.19, p = 0.03) when compared to patients with mutant IDH1. However, in our study population, MGMT methylation was not significantly associated with survival (HR = 1.38, p = 0.62) (Fig 5). IDH1 mutant GBM patients had a trend towards improved survival (HR = 7.9, p = 0.06) (Fig 5). These findings may be related to the modest number of total cases and the low percentages of MGMT-methylated and IDH1-mutant cases. RPS11 expression and age at diagnosis were significantly associated with survival (Fig 5). Patients diagnosed after the age of 60 exhibiting a 4-fold increase in their death hazard, (HR = 4.44, p = 0.02) (Fig 5). Multivariate analysis was not meaningful given the relatively small number of cases.

### Elevated transcription levels of RPS11 and RPS20 together are strongly associated with poor survival in a large TCGA dataset of primary glioblastoma

To confirm the prognostic value of RPS11, we expanded our investigation to gene expression (mRNA) levels and their correlation with survival of primary GBM patients in publicly available TCGA transcription data (n = 578 GBM) (https://tcga-data.nci.nih.gov/tcga/) (Table 4). Patients with RPS11 mRNA expression higher than average had a tendency towards a 20% increase in the hazard of death when compared with patients with lower than average RPS11 expression (95%CI: 0.99–1.43, p = 0.06). In a continuous expression analysis, we observed a 19% increase in the hazard of death with increased expression of RPS20 gene (95%CI: 1.04–1.51 p = 0.01). In a binned analysis, patients with higher than average RPS20 expression had a trend towards a 25% increase in the hazard of death compared to patients with lower than average RPS20 expression (p = 0.02). Patients with either overexpressed RPS11 or overexpressed RPS20 had a trend towards a 20% increase in the hazard of death relative to those with low RPS11 or RPS20 (95% CI: 0.97–1.49, p = 0.09). Strikingly, patients with both RPS11 and RPS20 overexpression exhibited a 43% increase in the hazard of death (95% CI: 1.11–1.83, p = 0.01) relative to those with low RPS11 and RPS20 expression. In the IDH1 copy-number variation (CNV)-adjusted analyses, results remain substantially unchanged. On the other hand, in both the IDH1 CNV and MGMT methylation status-adjusted analyses, similar results were obtained except the association between RPS11 mRNA expression and patient survival was not seen (Table 4). However, we should note that considering MGMT methylation depletes the original TCGA sample to only 290 subjects with available methylation data. We did not find any association between VEGFA mRNA expression and patient survival (Table 4).

**Table 4:** TCGA analysis—correlation with poor survival in newly diagnosed primary GBMs.

Biomarker(s)	Gene expression TCGA		
	Univariate Analysis	IDH1 Adjusted Estimates	IDH1 and MGMT Adjusted Estimates
	HR (95% CI) [P value]	HR (95% CI) [P value]	HR (95% CI) [P value]
**RPS11**	1.19 (0.99, 1.44) [0.06]	1.19 (0.99, 1.44) [0.06]	1.01 (0.77, 1.32) [0.96]
**RPS20**	1.25 (1.03, 1.50) [0.02]	1.24 (1.03, 1.50) [0.02]	1.35 (1.03, 1.77) [0.03]
**RPS11 or RPS20**	1.19 (0.97, 1.46) [0.09]	1.17 (0.94, 1.46) [0.15]	1.19 (0.87, 1.63) [0.29]
**RPS11 and RPS20**	1.43 (1.11, 1.83) [0.01]	1.42 (1.11, 1.83) [0.01]	1.31 (0.91, 1.90) [0.14]
**VEGFA**	1.12 (0.93, 1.35) [0.2]	1.13 (0.93, 1.36) [0.21]	1.10 (0.83, 1.46) [0.50]

## Discussion

In this study, we evaluated whether molecular defense signatures of TRGC could be used to identify biomarkers predictive of patient survival. We investigated both transcriptional and protein levels. Our strategy was to screen TRGC gene expression defense signatures using Probe Set Analyzer as an initial step to select candidate genes with prognostic value. This was followed by immunohistochemical analysis of GBM TMA and whole GBM tissues for the verification at the protein level, and finally correlation with a TCGA gene expression dataset. (S2A Fig). This proof-of-concept investigation of the prognostic value of TRGC markers demonstrates that TRGC defense signatures can be translated to patient prognosis. The results also suggest that TRGC are a clinically relevant model of the cell population within a GBM tumor that confers treatment resistance and facilitates development of tumor recurrence. Although increased RPS11 protein expression levels in either primary or secondary GBM tumors were associated with an increased hazard of death (Primary: HR = 3.95, p = 0.005; Secondary: HR = 6.85, p = 0.05), the prognostic significance was markedly enhanced when analysis was performed in newly diagnosed GBM (HR = 9.29, p<0.001) or newly diagnosed primary GBM (HR = 11.46, p<0.001). By contrast, the prognostic significance of RPS20 protein expression was only detected in primary GBM (HR = 2.58, p<0.02) or newly diagnosed primary GBM (HR = 4.51, p = 0.03). Concurrent increased expression of RPS11 and RPS20 in patients with newly diagnosed primary GBM showed an 18-fold increased hazard of death. This observation was also supported by the TCGA dataset, which showed that newly diagnosed primary GBM patients with both RPS11 and RPS20 overexpression exhibited a 43% increase in the hazard of death when compared to 19% and 25% increases for RPS11 and RPS20 individually. These data therefore support the view that upregulation of both RPS11 and RPS20 expression in patients with newly diagnosed primary GBM most robustly predicts a poor prognosis.

While RPS11 protein levels alone is a strong prognostic factor in newly diagnosed GBM or newly diagnosed primary GBM, elevated transcriptional levels only closely approached significance in the TCGA gene expression dataset (HR = 1.19, p = 0.06). This discrepancy may be due to the relative selectivity of immunohistochemical scoring for tumor cells and the stability of proteins, while global gene expression measurements are susceptible to perturbation by preanalytical variables and by intermixed non-neoplastic neuroglial, vascular, and inflammatory cells. Moreover, increased RPS11 protein level is associated with known unfavorable prognostic markers- unmethylated MGMT and wild-type IDH1 status, consistent with the premise that measurement of RP protein levels is clinically relevant. RPs are a major component of ribosomes that coordinate protein biosynthesis in the cytoplasm of cells. However, accumulated evidence from early studies have demonstrated that RPs have extraribosomal roles including cell cycle checkpoint, cell survival, malignant transformation, cell apoptosis/death, DNA repair, transcription, RNA processing, and anti-inflammatory functions [41–44]. Upregulation of transcripts for RPs in various types of human malignancies has been reported [45–48]. Increased expression of RPs in tumors are correlated with clinical stage and associated with poor survival [49, 50], suggesting the possibility that excess RP production in tumors may predict increased stress resistance to standard treatment. The expression of RPS11 protein was barely detected in normal colon mucosa but was upregulated in immature mucosal epithelium located in the crypt base and in colorectal carcinoma cells [46]. Importantly, it was shown that the expression of RPS11 does not significantly change in either human fibroblasts or peripheral blood mononuclear cells when subjected to a serum or mitogen stimulation, whereas RPS11 mRNAs rapidly decrease in HL60 leukemia cells induced to terminal differentiation by retinoic acid [51]. Moreover, RPS11 was shown to be specifically downregulated in apoptotic breast carcinoma cells [52]. These observations support the notion that upregulation of RPS11 may promote cells to remain in a dedifferentiated state that in turn renders them relatively resistant to environmental stress.

Recently, multiple lines of evidence have shown that a number of RPs are mouse double minute 2 homolog (MDM2)-binding partners, and it is suggested that, under nucleolar stress conditions, free RPs are released to the nucleoplasm where they interact with MDM2, leading to blockage of its E3 ligase function and resulting in the stabilization and activation of p53 [52–55]. A recent study has shown that nutrient deprivation inhibited ribosomal RNA biosynthesis and increased RP-MDM2 interaction, an acute stress response, leading to p53 activation and induction of p53-mediated transactivation of malonyl-CoA decarboxylase, thereby stimulating fatty acid oxidation, a catabolic pathway in response to nutrient depletion [56]. In concordance with this finding, we also found an association of treatment resistance with energy stress in TRGC that favor lipid catabolism as their major energy source [25]. Thus, upregulation of RPS11 in quiescent TRGC or in tumors may be associated with maintenance of a stem-like, stress-resistant phenotype. This may also explain that the association of upregulation of RPS11 with poor prognosis is more pronounced in patients with newly diagnosed primary GBM, a GBM subtype that more often lacks p53 mutations than secondary GBM [57, 58]. Moreover, prognostic significance of RPS11 was shown in newly diagnosed GBM but not in recurrent tumors, suggesting that RPS11 upregulation is of benefit to GBM tumor cells at initial treatment when radiochemotherapy is given to essentially all newly diagnosed GBM patients. Post-radiochemotherapy and after further evolution, it may be that recurrent GBM have acquired genetic or epigenetic changes that are biologically more important for outcome than RPS11. Like RPS11, RPS20 is one of the RPs that can also bind to MDM2 and activate p53 [59] and it was reported that overexpression of RPS20 is associated with an adverse outcome in medulloblastoma [60]. Analysis with STRING Interaction Network (http://string-db.org/), a tool that shows known and predicted protein-protein interactions, indicates that RPS20 directly binds to RPS11 (S2B Fig). Besides MDM2, two critical proteins that are associated with stress resistance—superoxide dismutase (SOD1) and ubiquitin C (UBC)-, are also linked with the RPS11-RPS20 protein network (S2B Fig). SOD1 is a vital detoxifying enzyme and a major antioxidant system, and is considered an anti-aging factor [61, 62]. Likewise, UBC is typically referred to as a stress-inducible gene that can adequately increase expression levels when cells are challenged with different types of stress; presumably to correct protein misfolding or to degrade damaged proteins allowing them to survive under toxic stress conditions [63–65]. These findings suggest that upregulation of RPS11 and RPS20 contributes to an anti-stress phenotype by enhancing genomic and cellular stability.

In this study, VEGFA, a factor targeted by bevacizumab therapy, is associated with poor prognosis only in newly diagnosed GBM or secondary GBM but not in primary GBM alone. Similarly, VEGFA analysis of the TCGA dataset where primary GBM were evaluated did not show an association with prognosis. Probe Set Analyzer did not have data for secondary GBM to compare against our protein data. Future study of this GBM subset is desirable. In contrast, RPS11 and/or RPS20 are robust prognostic factors. Targeted therapy that aims to reduce these RPs may potentially reverse the stress-resistant phenotype and sensitize tumors to standard radiochemotherapy. Given their ubiquity, targeted therapy against RPs without adversely affecting normal cells is likely to prove challenging. Identification of unique transcriptional inducers that promote the overexpression of RPS11 and RPS20 in GBM could facilitate elucidation of the underlying biology and identification of additional prognostic factors or treatment targets.

This study strongly supports the concept of TRGC as a useful cellular model and a resource for identifying novel GBM prognostic biomarkers. We have found that overexpression of RPS11 and RPS20 can predict decreased survival in patients with newly diagnosed primary GBM at either the transcriptional or protein levels. RPS11 and RPS20 together have greater prognostic value than VEGFA, a major target of current therapy, suggesting that these RPs are worthwhile targets of novel therapeutic strategies.

## Supporting Information

S1 Fig Additional representative outputs of Probe Set Analyzer for selected molecular signatures of TRGC. (DOCX)

S2 Fig Schematic diagram illustrating the essential steps and a screening process for identification of TRGC-based prognostic factors for patients with glioblastoma. (DOCX)

S1 Table Molecular signatures of TRGC determined in patient tumors associated with poor prognosis. (DOCX)

S2 Table Tissue microarray protein expression- Cox proportional hazard analysis of secondary GBM. (DOCX)
