# Supplementary material for: Ribosomal Proteins RPS11 and RPS20, Two Stress-Response Markers of Glioblastoma Stem Cells, Are Novel Predictors of Poor Prognosis in Glioblastoma Patients
Source: PLoS One. 2015 Oct 27;10(10):e0141334. doi: 10.1371/journal.pone.0141334 (PMC4624638; doi:10.1371/journal.pone.0141334)
Supplement: S1 Fig — (DOCX) [file pone.0141334.s001.docx]

**Figure S1.** Additional representative outputs of the Probe Set Analyzer for selected molecular signatures of TRGC that are associated with patient prognosis (also see Figure 2 in text).
