# Supplementary material for: Ribosomal Proteins RPS11 and RPS20, Two Stress-Response Markers of Glioblastoma Stem Cells, Are Novel Predictors of Poor Prognosis in Glioblastoma Patients
Source: PLoS One. 2015 Oct 27;10(10):e0141334. doi: 10.1371/journal.pone.0141334 (PMC4624638; doi:10.1371/journal.pone.0141334)
Supplement: S2 Fig — (DOCX) [file pone.0141334.s002.docx]

**Figure S2. Schematic diagram illustrating the essential steps and a screening process for identification of TRGC-based prognostic factors for patients with glioblastoma.**  A. Glioblastoma stem cell (GSC) culture lines were established from fresh tumors. Treatment-sensitive GSC clones (TSGC) and treatment-resistant GSC clones (TRGC) were selected from GSC cultures. The tumorigenicity of sensitive and resistant clones was verified by injecting live cells into mice intracranially. Defense biomarkers of TRGC were identified via a comparative microarray analysis between TSGC and TRGC. The correlation between selected defense markers and prognosis in patients with glioblastoma was established through the indicated immunohistochemical and gene expression screening. B. Proteins interactions with RPS11 are depicted using the STRING Interaction Network tool. The network nodes are proteins. Nodes are either colored if they are directly linked to RPS11 or white if they are nodes of a higher iteration/depth. Different line colors represent the types of evidence for associations.
