# Supplementary material for: Ribosomal Proteins RPS11 and RPS20, Two Stress-Response Markers of Glioblastoma Stem Cells, Are Novel Predictors of Poor Prognosis in Glioblastoma Patients
Source: PLoS One. 2015 Oct 27;10(10):e0141334. doi: 10.1371/journal.pone.0141334 (PMC4624638; doi:10.1371/journal.pone.0141334)
Supplement: S1 Table — (DOCX) [file pone.0141334.s003.docx]

**Table S1. Molecular signatures of TRGC determined in patient tumors associated with poor prognosis***

|  | **Newly diagnosed**  **tumors**  **p value (LFDR)** | **Recurrent**  **Tumors**  **p value (LFDR)** |
| --- | --- | --- |
| **ENPP2: ectonucleotide pyrophosphatase/phosphodiesterase 2**  **TXNIP: thioredoxin interacting protein**  **SUPT16H: suppressor of Ty 16 homolog (S. cerevisiae)**  **EGR1: early growth response 1**  **SSFA2: sperm specific antigen 2***  **COL6A2: collagen, type VI, alpha 2**  **IL6ST: interleukin 6 signal transducer (gp130)**  **EPRS: glutamyl-prolyl-tRNA synthetase**  **MFAP4: microfibrillar-associated protein 4**  **VEGFA: vascular endothelial growth factor A**  **NFIX: nuclear factor I/X***  **ALDH3A2: aldehyde dehydrogenase 3 family, member A2***  **FBN1: fibrillin 1**  **ZC3H11A: zinc finger CCCH-type containing 11A**  **PLD3: phospholipase D family, member 3**  **FMNL2: formin-like 2**  **TPR: translocated promoter region (to activated MET)**  **PPFIBP1: PTPRF interacting protein, binding protein 1**  **RPS11: Ribosomal protein S11**  **TPM4: tropomyosin 4**  **SLC38A1: solute carrier family 38, member 1**  **RPS20: ribosomal protein S20**  **PSAT1: phosphoserine aminotransferase 1**  **PPP1R3C: protein phosphatase 1, regulatory subunit 3C**  **RPL38: ribosomal protein L38**  **MAP4: microtubule-associated protein 4**  **PRKCI: protein kinase C, iota**  **RPL27A: Ribosomal protein L27a**  **HNRPA3: heterogeneous nuclear ribonucleoprotein A3**  **OSBPL8: oxysterol binding protein-like 8**  **RAD23A: RAD23 homolog A (S. cerevisiae)**  **FOLR1: folate receptor 1 (adult)** | **0.34 (0.165)**  **0.0074 (0.020)**  **0.029 (0.029)**  **0.0034 (0.004)**  **0.014 (0.029)**  **0.36 (0.165)**  **0.19 (0.094)**  **0.6 (0.188)**  **1.9e-10 (<0.001)**  **0.66 (1.0)**  **0.0021 (0.002)**  **0.0027 (0.002)**  **7.7e-08 (<0.001)**  **4.9e-12 (<0.001)**  **0.18 (0.094)**  **0.6 (0.188)**  **0.22 (0.165)**  **2.5e-05 (0.001)**  **5.8e-07 (<0.001)**  **5e-04 (0.001)**  **0.58 (0.165)**  **0.0065 (0.004)**  **0.19 (0.094)**  **0.21 (0.094)**  **0.0066 (0.004)**  **0.67 (1.0)**  **0.17 (0.094)**  **0.003 (0.002)**  **0.00095 (0.002)**  **0.47 (0.165)**  **0.41 (0.165)**  **0.033 (0.094)** | **0.013 (0.023)**  **0.88 (1.0)**  **0.21 (0.866)**  **0.52 (1.0)**  **0.44 (0.866)**  **0.00073 (0.023)**  **7.4e-05 (<0.001)**  **0.02 (0.1)**  **0.72 (1.0)**  **2.5e-07 (<0.001)**  **0.57 (1.0)**  **0.00015 (0.001)**  **0.65 (1.0)**  **0.016 (0.023)**  **0.0091 (0.023)**  **3.4e-09 (<0.001)**  **3e-04 (0.002)**  **8.5e-05 (0.001)**  **0.32 (0.866)**  **0.00048 (0.002)**  **0.032 (0.125)**  **1.6e-06 (<0.001)**  **0.047 (0.681)**  **0.00024 (0.001)**  **0.14 (0.681)**  **0.02 (0.1)**  **1.2e-06 (<0.001)**  **0.0096 (0.023)**  **0.011 (0.023)**  **3.2e-0.5 (<0.001)**  **2.9e-10 (<0.001)**  **3.6e-07 (<0.001)** |

*Selected molecular signatures of treatment–resistant glioblastoma stem cell clones (TRGC) that are detected

in patients' biopsies or excised tumor tissues, are associated with patient survival as determined by the Probe

Set Analyzer. Multiplicity-adjusted calculations of local false discovery rates (LFDR) = 5%, and LFDR

<0.05 is considered significant.
