# Supplementary material for: Ribosomal Proteins RPS11 and RPS20, Two Stress-Response Markers of Glioblastoma Stem Cells, Are Novel Predictors of Poor Prognosis in Glioblastoma Patients
Source: PLoS One. 2015 Oct 27;10(10):e0141334. doi: 10.1371/journal.pone.0141334 (PMC4624638; doi:10.1371/journal.pone.0141334)
Supplement: S2 Table — (DOCX) [file pone.0141334.s004.docx]

**Table S2. Tissue microarray protein expression- Cox proportional**

**hazard analysis of secondary GBM**

| **Marker** | **Secondary GBM- High vs. Low** HR (95%CI) [p value] |
| --- | --- |
| **VEGFA** | 4.9(1.33-18.06)[0.05] |
| **RPS11** | 6.85(1.36-34.36)[0.05] |
| **RPS20** | 0.52(0.08-4.14)[0.54] |
